# Prevalence of potential underlying aetiology of macrocytic anaemia in Dutch general practice

**DOI:** 10.1186/s12875-016-0514-z

**Published:** 2016-08-19

**Authors:** Karlijn Stouten, Jurgen A. Riedl, Jolanda Droogendijk, Rob Castel, Joost van Rosmalen, Ron J. van Houten, Paul Berendes, Pieter Sonneveld, Mark-David Levin

**Affiliations:** 1 Department of Clinical Chemistry, Albert Schweitzer Hospital, Albert Schweitzerplaats 25, room F3126, 3300 AK Dordrecht, The Netherlands; 2 Department of Internal Medicine, Albert Schweitzer Hospital, Albert Schweitzerplaats 25, 3300 AK Dordrecht, The Netherlands; 3 Department of Haematology, St Elisabeth Hospital, Hilvarenbeekse Weg 60, 5022 GC Tilburg, The Netherlands; 4 Department of Clinical Chemistry, Admiraal de Ruyter Hospital, ‘s-Gravenpolderseweg 114, 4462 RA Goes, The Netherlands; 5 Department of Biostatistics, Erasmus Medical Centre, ’s-Gravendijkwal 230, 3015 CE Rotterdam, The Netherlands; 6 General practitioner at General Medical Practice van Houten, Tromplaan 49, 3342 TR, H.I., Ambacht, The Netherlands; 7 Department of Clinical Chemistry, Beatrix Hospital, Banneweg 57, 4204 AA Gorinchem, The Netherlands; 8 Department of Haematology, Erasmus Medical Centre, ‘s-Gravendijkwal 230, 3015 CE Rotterdam, The Netherlands

**Keywords:** Erythrocytes, Erythropoiesis, Macrocytic anaemia, General practice, Survival, Laboratory markers

## Abstract

**Background:**

Macrocytic anaemia (MCV ≥ 100 fL) is a relatively common finding in general practice. However, literature on the prevalence of the different causes in this population is limited. The prevalence of macrocytic anaemia and its underlying aetiology were analysed in a general practice population. The potential effect of the different aetiology on survival was also evaluated.

**Methods:**

Between the 1st of February 2007 and the 1st of February 2015, patients aged 50 years or older and presenting to their general practitioner with a newly diagnosed anaemia, were included in the study. Anaemia was defined as haemoglobin level below 13.7 g/dL in men and below 12.1 g/dL in women. A broad range of laboratory tests was performed for each patient. The causes of anaemia were consequently determined by two independent observers based on the laboratory results.

**Results:**

Of the 3324 included patients, 249 (7.5 %) displayed a macrocytic anaemia and were subsequently analysed. An underlying explanation could be established in 204 patients (81.9 %) with 27 patients (13.2 %) displaying multiple causes. Classic aetiology (i.e. alcohol abuse, vitamin B12/folic acid deficiency, haemolysis and possible bone marrow disease) was found in 115 patients. Alternative causes (i.e. anaemia of chronic disease, iron deficiency, renal anaemia and other causes) were encountered in 101 patients. In addition, a notable finding was the median gamma GT of 277 U/L in patients diagnosed with alcohol abuse (*N* = 24, IQR 118.0-925.5) and 23 U/L in the remaining cohort (*N* = 138, IQR 14.0-61.0). The distribution of gamma GT values was statistically different (*P* < 0.001).

Five year survival rates were determined for six categories of causes, ranging from 39.9 % (95 % CI 12.9-66.9) for renal anaemia to 76.2 % (95 % CI 49.4-103.0) for the category multiple causes.

**Conclusion:**

In addition to classic explanations for macrocytosis, alternative causes are frequently encountered in patients with macrocytic anaemia in general practice.

## Background

Anaemia is regularly encountered in general practice and hospital settings. In a community dwelling population, the prevalence of anaemia starts increasing after age 50, starting from about 5.5 % in the age group 50-64 years and rising to over 20 % in the age group 85 and older [1]. Anaemia has been shown to be an independent predictor of survival in a population of community-dwelling older adults and is capable of negatively influencing quality of life [2, 3].

Macrocytic anaemia (mean corpuscular volume (MCV) ≥ 100 fL) is a relatively common finding. Prevalence estimates for macrocytosis range from 1.7 % to 3.9 % [4, 5]. About 40 % of patients presenting with macrocytosis display an associated anaemia [4, 5]. Classic causes of macrocytosis include nutrient deficiencies (i.e. vitamin B12 and folic acid), alcohol abuse, liver disease, haemolysis, medication and primary bone marrow disease (e.g. myelodysplastic syndrome (MDS)) [3–6]. Alternative causes include iron deficiency anaemia (IDA), anaemia of chronic disease (ACD) and renal anaemia, with prevalence ranges of 13.6-47.2 % [7], 16.0-19.0 % [8–10] and 10.5-19.7 % [9–12], respectively. However, the prevalence of these causes in patients with macrocytic anaemia remains to be determined.

A cohort study was set up to systematically evaluate patients presenting with anaemia to their general practitioner. Since literature on macrocytic anaemia in general practice is limited, this study cohort was used to clarify the prevalence of macrocytic anaemia and its aetiology in newly diagnosed anaemia patients in general practice. Besides considering the classical causes of macrocytosis, the prevalence of laboratory abnormalities associated with IDA, ACD and renal anaemia was evaluated, since these have not been described in a macrocytic cohort before. The potential impact of the different aetiology on survival was also assessed.

## Methods

### Patient inclusion

This study was approved by the ethical committee of the Albert Schweitzer Hospital and was part of a large transmural project aimed at anaemic general practice patients aged 50 years and older. The main goal of this project is to improve care provided for anaemic patients. The study was conducted in the Western part of the Netherlands, and included 63 participating general practitioners. Patients presented to a general practitioner (GP) displaying symptoms indicative of anaemia (e.g. fatigue, dizziness, pallor or general malaise). If laboratory tests established a newly diagnosed anaemia (i.e. no known anaemia the preceding two years), a standardised laboratory work-up was immediately performed. Anaemia was defined as haemoglobin level below 13.7 g/dL (8.5 mmol/L) for men and below 12.1 g/dL (7.5 mmol/L) for women. These levels constitute the cut-off values recommended by the Dutch College of General Practitioners (DCGP) [13]. Patients were included between the 1st of February 2007 and the 1st of February 2015. The follow-up period ended on the 1st of March 2016. From this anaemic cohort, all patients with a macrocytic anaemia (MCV ≥ 100 fL) were selected for further analysis.

### Laboratory work-up and data collection

For each patient, a standardised laboratory work-up was performed, including haemoglobin, MCV, erythrocytes, erythrocyte sedimentation rate, reticulocytes, thrombocytes, leukocytes, ferritin, transferrin, serum iron, serum vitamin B12, serum folic acid, gamma GT, lactate dehydrogenase (LDH), creatinine and C-reactive protein (CRP). This work-up allowed for the consideration of a broad range of causes of anaemia. The results for gamma GT and creatinine were not complete for all patients. Due to retrospective data collection, these values could not be recovered. At the discretion of the general practitioner, patients were referred to the hospital for additional diagnostic testing. For these patients, a hospital chart review was conducted and any additional examinations were analysed.

### Classification of causes of macrocytic anaemia

A classification system was developed, which included the following causes: 1) vitamin B12 deficiency 2) folic acid deficiency 3) possible bone marrow disease 4) haemolysis 5) documented alcohol abuse 6) anaemia of chronic disease (ACD) 7) iron deficiency anaemia (IDA) 8) renal anaemia and 9) other. If no cause could be determined, it was classified as unknown. See Table 1 for the definition of each cause.

**Table 1:** Definitions of the different causes found in the macrocytic cohort

Cause	Definition
1) Vitamin B12 deficiency	Vitamin B12 < 130 pmol/L
2) Folic acid deficiency	Folic acid < 5 nmol/L
3) Possible bone marrow disease	Abnormal number leukocytes and thrombocytes Reticulocytes < 2.5 %
4) Haemolysis	Raised LDH, reticulocytes > 2.5 %, bilirubin > 17 μmol/L
5) Documented alcohol abuse	Documented in patient file by treating physician
6) Anaemia of chronic disease (ACD)	Ferritin > 100 μg/L, serum transferrin < 2.0 g/L and/or serum iron < 14 μmol/L (men), < 10 μmol/L (women)
7) Iron deficiency anaemia (IDA)	Ferritin < 25 μg/L (men), < 20 μg/L (women)
8) Renal anaemia	Estimated creatinine clearance (MDRD) ≤ 45 mL/min/1.73 m^2 (8)^
9) Other	Documented in patient file by treating physician
10) Unknown	No cause could be determined

These cut-off values were derived from the reference intervals used in the Clinical Chemistry laboratory of the Albert Schweitzer Hospital where all tests were conducted

Regularly, patients diagnosed with renal anaemia also displayed features of ACD. These features were considered due to the present renal anaemia, and therefore, ACD was not classified as a separate cause in these cases. The cause of anaemia was established by two independent observers (authors KS and ML, anaemia researcher and haematologist, respectively). In case of discordance, the observers deliberated until a consensus was reached. Patients with missing creatinine values could not be considered for the cause renal anaemia.

The possible association of the patient’s age group (50-64 years, 65-74 years, 75-84 years and 85+ years) and gender with the diagnosed cause of macrocytic anaemia was also analysed. Vitamin B12 deficiency, folic acid deficiency and iron deficiency were combined into the cause ‘nutrient deficiency’ for analysis.

### Factors influencing survival

Survival data was extracted from the hospital and laboratory information system. Patients were followed until either their deaths, or until the 1st of March 2016, at which moment they were censored at the last date they were documented as alive in the hospital or laboratory information system.

Patients were classified into six groups for survival analysis; nutrient deficiency (vitamin B12 deficiency, folic acid deficiency and iron deficiency), ACD, renal anaemia, multiple causes (patients who presented with a double or triple cause, except those presenting with only nutrient deficiencies), other (haemolysis, possible bone marrow disease, documented alcohol abuse and other) and unknown.

### Statistics

Continuous variables were described using medians and interquartile ranges (IQR), and categorical variables were described using percentages. Fisher’s exact tests were used to compare the relative prevalence (among all patients with macrocytic anaemia) of causes of anaemia between men and women and between different age groups. This test was chosen to account for the very small number of cases in some comparisons. These tests were performed separately for each cause of anaemia (vitamin B12, folic acid and iron deficiency combined into nutrient deficiency) and for each pair of age groups. The Mann-Whitney test was used to compare the distribution of gamma GT values between patient groups. Kaplan-Meier analysis and the log rank test were used for a univariate analysis of the association between causes of anaemia and survival. A Cox proportional-hazards model correcting for the cause of anaemia, age (coded as a continuous variable), gender, date of inclusion, and haemoglobin (divided into mild (Hb ≥ 10.0 g/dL), moderate (Hb 8.0-9.9 g/dL) and severe (Hb < 8.0 g/dL) anaemia) level was used for a multivariate analysis of survival. The proportional hazards assumption in this model was tested by including interaction effects of the independent variables with time in a Cox model with time-dependent covariates. To account for the effects of multiple testing, a Bonferroni-adjusted significance level of 0.006 was used for Fisher’s exact tests and a level of 0.008 was used for log-rank tests. For all other tests, a two-sided significance level of 0.05 was used. Calculations were performed using Statistical Package for Social Sciences (SPSS) version 18 for Windows.

## Results

Over the span of eight years, a total of 3324 patients with newly diagnosed anaemia were included in the study, of whom 249 (7.5 %) patients presented with a macrocytic anaemia; 140 men (median age 75 years, IQR = 63-82 years) and 109 women (median age 80 years, IQR = 72.5-87 years). An overview of the patient characteristics can be found in Table 2.

**Table 2:** Patient characteristics of 249 patients with macrocytic anaemia

Parameters	Median: IQR	Reference values
Age	78.0 years: 65.0-85.0	
- Men	75.0 years: 63.0-82.0	
- Women	80.0 years: 72.5-87.0	
Haemoglobin	11.8 g/dL: 10.5-12.9	
- Men	12.9 g/dL: 11.3-13.2	13.7-17.7 g/dL
- Women	11.3 g/dL: 10.2-11.8	12.1-16.1 g/dL
MCV	103 fL: 101-108	
Erythrocytes	3.5 10^12/L: 3.0-3.8	
- Men	3.8 10^12/L: 3.1-4.0	4.6-6.2 10^12/L
- Women	3.3 10^12/L: 2.9-3.6	4.2-5.4 10^12/L
Reticulocytes	1.3 %: 0.9-1.8	<2.5 %
Thrombocytes	218.0 10^9/L: 166.0-272.5	150-400 10^9/L
Leukocytes	6.7 10^9/L: 5.0-8.7	4.3-10.0 10^9/L
Ferritin	225.0 μg/L: 131.5-464.0	
- Men	250.5 μg/L: 137.0-557.8	25-250 μg/L
- Women	197.0 μg/L: 107.0-385.0	20-150 μg/L
Creatinine*	84.0 μmol/L: 70.0-105.5	
- Men	88.0 μmol/L: 72.5-105.0	59-104 μmol/L
- Women	76.5 μmol/L: 62.0-106.0	45-84 μmol/L
Gamma GT*	27.5 U/L: 16.0-112.5	
- Men	29.5 U/L: 18.0-168.5	<50 U/L
- Women	23.0 U/L: 14.0-95.0	<35 U/L

The interquartile range (IQR) is denoted by the first and third quartile. *The number of patients with values for creatinine and gamma GT were 209 and 162 patients, respectively

A single cause of anaemia was established in 177 patients (71.1 %). A double cause was established in 26 patients (10.4 %) and a triple cause in one patient (0.4 %). A total number of 232 causes could be established in these 204 patients, while the cause remained unknown in 45 patients (18.1 %).

### Classic causes

**Fig. 1:**
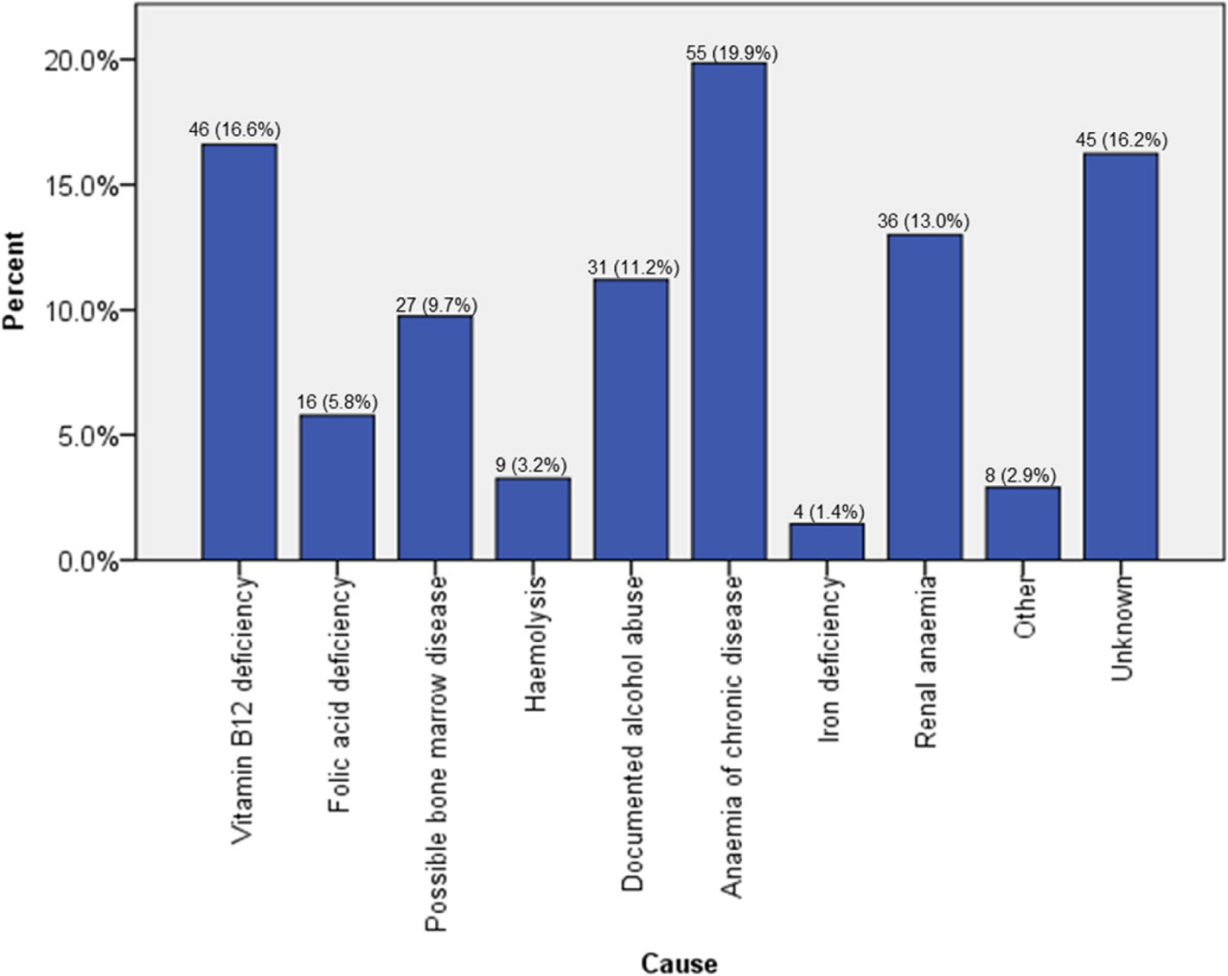
An overview of the frequency of each established cause in a macrocytic population. For each patient the cause or causes were diagnosed by two independent observers

In this cohort, 115 patients (46.2 %) presented with a total of 129 classic causes of macrocytic anaemia. Vitamin B12 deficiency was found 46 times and folic acid deficiency 16 times. Documented alcohol abuse was established 31 times, haemolysis 9 times and possible bone marrow disease 27 times (Fig. 1). Bone marrow examinations performed in this last category revealed seven cases of myelodysplastic syndrome (MDS), six cases of acute myeloid leukaemia, two cases of chronic lymphoid leukaemia and two cases of multiple myeloma. In one patient no pathology could be found. In six patients with possible bone marrow disease, no further examinations were performed due to their advanced age, while in the remaining three patients, the abnormal leukocytes and thrombocytes counts were deemed related to the concurrent nutrient deficiency.

### Alternative causes

A total of 103 alternative causes of macrocytic anaemia were found in 101 patients (40.6 %). IDA was encountered 4 times and ACD was found 55 times. Renal anaemia was established 36 times and other causes 8 times (Fig. 1). This last category included three patients presenting with gastrointestinal bleeding, and three patients diagnosed with hypothyroidism. One patient was diagnosed with liver cirrhosis and one patient presented shortly after undergoing surgery.

### Unknown cause of anaemia

Forty-five patients presented with an unknown cause for their anaemia. Two patients underwent a bone marrow examination in an attempt to elucidate the underlying aetiology. One patient was subsequently diagnosed with MDS. For the second patient, the cause of anaemia remained unknown.

### Factors influencing diagnosis

*Age* 58, 40, 85 and 66 patients were found in the age groups 50-64 years, 65-74 years, 75-84 years and 85+ years, respectively.

**Fig. 2:**
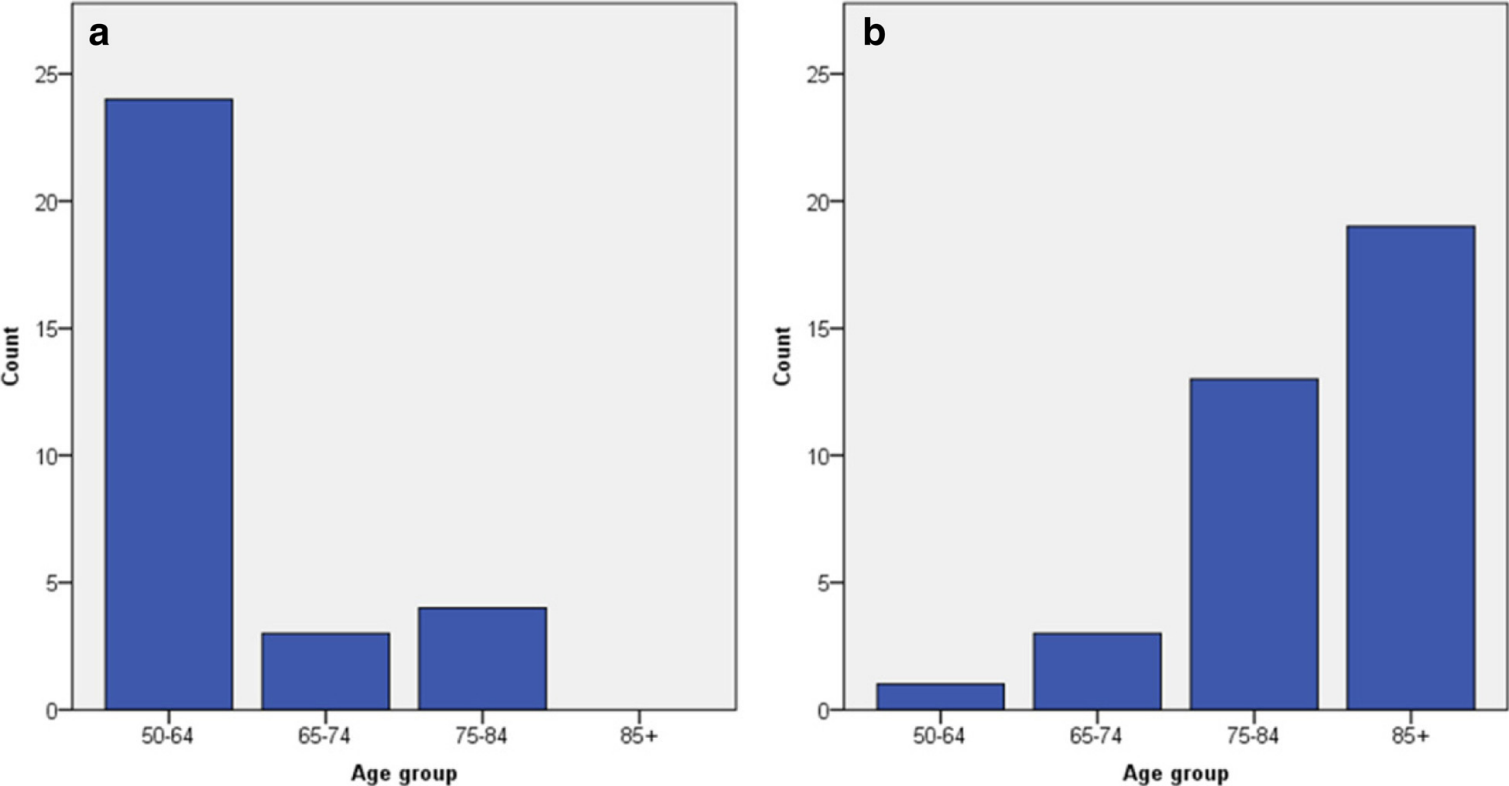
Number of cases per age group for the causes documented alcohol abuse (**a**) and renal anaemia (**b**)

Documented alcohol abuse (Fig. 2-a) was most often found in the age group 50-64 years (24 of the 31 cases (77.4 %)). Significant differences were found between this age group and the other three groups (*P* < 0.001 for all comparisons). Increased gamma GT is often associated with alcohol abuse. The median gamma GT value of patients diagnosed with alcohol abuse was 277.0 U/L (*N* = 24, IQR 118.0-925.5). The remaining cohort showed a median value of 23.0 U/L (*N* = 138, IQR 14.0-61.0). The distribution of gamma GT values between these two groups was statistically different (*P* < 0.001).

Renal anaemia (Fig. 2-b) showed a rising prevalence per age group from 1 case in 50-64 years (1.7 %) to 19 cases in 85+ years (28.8 %). For this cause, a significant difference was found between age groups 50-64 years and 85+ years (*P* < 0.001).

*Gender* Renal anaemia was diagnosed more often in women compared to men, in 22 of 109 (20.2 %) and 14 of 140 (10.0 %) patients, respectively, but this difference was not significant (*P* = 0.019). No other distinct observations were made when analysing the association of gender with the cause of anaemia.

### Factors influencing survival

**Fig. 3:**
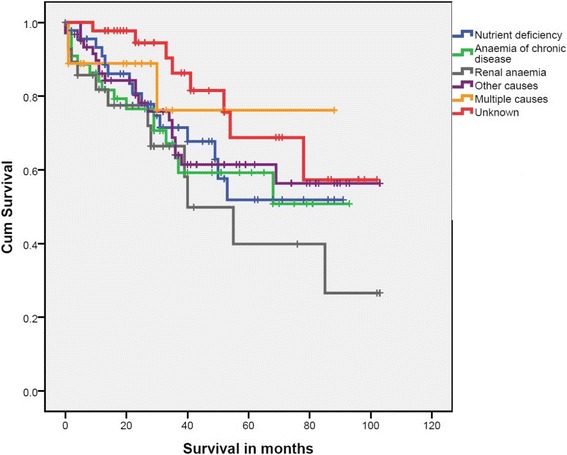
Kaplan-Meier curves of the survival from the moment of inclusion for the six main causes included in survival analysis. The log-rank test showed no significant differences in survival between causes (P = 0.192)

The median follow-up period lasted 29 months (95 % CI 25-32 months, IQR 14-52). The Kaplan-Meier estimate of five-year survival rate from the moment of inclusion was 58.8 % (95 % CI 50.4-67.2) for the overall population, with a rate of 58.7 % (95 % CI 48.1-69.3) for men and a rate of 58.4 % (95 % CI 44.2-72.6) for women. Five-year survival rates were also determined for the six groups used for survival analysis: nutrient deficiency 51.9 % (95 % CI 31.7-72.1), anaemia of chronic disease 59.2 % (95 % CI 42.0-76.4), renal anaemia 39.9 % (95 % CI 12.9-66.9), other causes 61.4 % (95 % CI 47.2-75.6), multiple causes 76.2 % (95 % CI 49.4-103.0) and unknown 68.8 % (95 % CI 47.6-90.0) (See Fig. 3). The log rank test revealed no significant differences in survival between these groups (*P* = 0.192).

The possible association of the six main causes with survival after entry into the study was analysed using a Cox proportional hazards model, correcting for age, gender, date of inclusion and haemoglobin level. The group ‘unknown’ was selected as reference since its median haemoglobin level was closest to the normal range (personal observation by author) and due to its size. However, no significant association of anaemia aetiology with survival was found (See Table 3). No significant violations of the proportional hazards assumption were detected.

**Table 3:** Results of Cox proportional-hazards model

	Hazard ratio	95 % CI	*P*-value
Cause			0.213
Nutrient deficiency	1.84	0.76-4.48	0.179
Anaemia of chronic disease	2.47	1.05-5.82	0.038
Renal anaemia	2.31	0.93-5.74	0.072
Other causes	2.94	1.27-6.82	0.012
Multiple causes	2.09	0.54-8.07	0.285
Unknown		Reference	
Severity (haemoglobin)			0.476
Moderate	1.41	0.64-3.12	0.396
Severe	0.62	0.19-2.04	0.433
Mild		Reference	
Gender			0.287
Female	0.77	0.47-1.25	0.287
Male		Reference	
Age (years)	1.06	1.03-1.09	<0.001
Date of inclusion (years)	0.927	0.82-1.04	0.213

The Cox proportional-hazards model analysing the possible association of the aetiology of macrocytic anaemia with survival after entry into the study, found no significant results for the different causes

## Discussion

To determine the prevalence of macrocytic anaemia and its causes, defined as associations with laboratory abnormalities, the macrocytic patients of a unique cohort of patients presenting to their general practitioner with newly diagnosed anaemia, were analysed. Both classic and alternative causes of macrocytic anaemia were regularly established in this cohort. To the best of our knowledge, this is the largest study of macrocytic anaemia patients in general practice to date.

Several algorithms have been developed to aid in diagnosing the underlying cause of macrocytic anaemia [3, 14–16]. ACD, IDA and renal anaemia are rarely considered causes of macrocytic anaemia by these algorithms, since these causes are usually reported in patients with a microcytic (MCV ≤ 80 fL) or normocytic (80 < MCV > 100) anaemia [15, 17, 18]. However, all three causes were frequently encountered in this macrocytic population. It is unclear what causes this shift towards macrocytosis in these patients. In literature, Andrews (2008) mentioned a possible tendency of the erythrocytes in ACD towards macrocytosis, but did not explain this further [19]. Furthermore, patients with IDA, ACD and renal anaemia were, on average, only mildly macrocytic compared to patients diagnosed with classical causes of macrocytosis, such as nutrient deficiency, alcohol abuse and possible bone marrow disease (personal observation by author).

For the purpose of this study, a strict limit was used when establishing the presence of vitamin B12 or folic acid deficiency. However, values measured just above these limits (range 131-200 pmol/L for vitamin B12 and 5-10 nmol/L for folic acid) may not necessarily exclude a deficiency. Several patients who were classified as having an ACD, IDA or renal anaemia, may have had an additional underlying deficiency of vitamin B12 or folic acid, which remained unclassified due to these strict limits. Other possible explanations for the presence of ACD, IDA and renal anaemia cases, include an undiscovered underlying alcohol abuse or undetected bone marrow disease. Certain medications can also cause macrocytosis. However, a complete record of medication was not available for each patient.

According to Younes et al. (2013), unexplained macrocytosis may be a prelude to a bone marrow disease; 11.6 % of their patients developed a bone marrow disease during the study period [5]. However, at the end of the follow-up period, only 1 of the 45 patients (2.2 %) presenting with unknown anaemia in this study was diagnosed with such disease, according to the documentation available in the hospital information system. The strict limits used for the classification of vitamin B12 and folic acid deficiency or undetected alcohol abuse, as described above, may also explain the presence of macrocytic anaemia in the 44 patients whose cause of anaemia remained unknown (the 45th patient with unknown anaemia was subsequently diagnosed with MDS). The MDRD value was unknown in nine patients in this category, leaving us unable to exclude the presence of renal anaemia.

Documented alcohol abuse was found most often among those aged 50-64 years. After age 65, the prevalence began to decrease. This is consistent with data from a large-scale study aimed at determining prevalence of alcohol abuse performed in the United States [20].

Renal anaemia showed a prevalence rising with age and was found significantly more often in patients older than 75 years. This may be explained by a decrease in renal function with age [21]. In addition, renal anaemia was observed more often among women. This may be due to the relatively high proportion of women in the two oldest age groups; 68.7 % of the included women were over 75 years old at the time of inclusion versus 45.0 % of men.

The possible association of different causes of anaemia with survival has not been studied before within a macrocytic cohort. However, no significant influence of the causes nutrient deficiency, renal anaemia, anaemia of chronic disease, other causes and multiple causes on survival could be found.

### Limitations

Due to the retrospective set-up of the study, it relied completely on patient records. Laboratory records were complete but records kept by the GP were inaccessible while hospital records were not available for each patient, resulting in an incomplete overview of medication, co-morbidity, alcohol abuse and follow-up diagnostics. Due to loss of follow-up the survival data were not complete for each patient. At-risk patients are more likely to visit the hospital or laboratory and therefore more likely to have complete survival data. This may have lowered the calculated survival rate. The median age of this population is high (78 years), which may make age too strong a confounding factor for this survival analysis.

## Conclusions

Macrocytic anaemia was found in 7.5 % of patients with a newly diagnosed anaemia in general practice. Notable findings were the high prevalence of alcohol abuse in the age group 50-64 years and the often multifactorial nature of anaemia in this elderly population (≥50 years). Laboratory abnormalities associated with anaemia of chronic disease, iron deficiency anaemia and renal anaemia were frequently encountered in this macrocytic cohort. However, current algorithms for the diagnosis of macrocytic anaemia generally do not consider these alternative causes. The different underlying causes of anaemia could have severe consequences should they remain undetected. A broad diagnostic work-up, without being led by the MCV, is therefore recommended to fully elucidate the underlying cause of macrocytic anaemia.

## Abbreviations

ACD: Anaemia of chronic disease
CRP: C-reactive protein
DCGP: Dutch College of general practitioners
GP: General practitioner
IDA: Iron deficiency anaemia
IQR: Interquartile range
LDH: Lactate dehydrogenase
MCV: Mean corpuscular volume
MDS: Myelodysplastic syndrome
SPSS: Statistical package for social sciences
